# Alterations of kynurenine pathway in alcohol use disorder and abstinence: a link with gut microbiota, peripheral inflammation and psychological symptoms

**DOI:** 10.1038/s41398-021-01610-5

**Published:** 2021-10-01

**Authors:** Sophie Leclercq, Markus Schwarz, Nathalie M. Delzenne, Peter Stärkel, Philippe de Timary

**Affiliations:** 1 Institute of Neuroscience, Université catholique de Louvain (UCLouvain), Brussels, Belgium; 2 Metabolism and Nutrition Research Group, Louvain Drug Research Institute, Université catholique de Louvain (UCLouvain), Brussels, Belgium; 3 Institute of Laboratory Medicine, LMU Klinikum Munich, Munich, Germany; 4 Laboratory of Hepato-Gastroenterology, Institute of Experimental and Clinical Research, Université catholique de Louvain (UCLouvain), Brussels, Belgium; 5 Department of Hepatogastroenterology, Cliniques universitaires Saint-Luc, Brussels, Belgium; 6 Department of Adult Psychiatry, Cliniques universitaires Saint-Luc, Brussels, Belgium

**Keywords:** Addiction, Physiology

## Abstract

The gut-brain communication is mostly driven by the immune, metabolic and neural pathways which remained poorly explored in patients with alcohol use disorder (AUD). The metabolites arising from the tryptophan-kynurenine pathway have gained considerable attention since they are at the interface between intestinal bacteria, host immune response and brain functions. This study described the circulating levels of kynurenine metabolites in AUD patients, at the onset (T1) and end (T2) of a 3-week detoxification program, and tested correlations between those metabolites and inflammatory markers, the gut microbiota and the psychological symptoms. Increased concentration of the neurotoxic metabolite quinolinic acid (QUIN) and decreased levels of the neuroprotector metabolite kynurenic acid (KYNA) which both modulate glutamatergic neurotransmission were observed in AUD patients, particularly at T2. The inflammatory marker hsCRP was associated with several metabolic ratios of the kynurenine pathway. Tryptophan, KYNA and QUIN were correlated with depression, alcohol craving and reaction time, respectively. Analysis of gut microbiota revealed that bacteria known as short-chain fatty acid producers, as well as bacterial metabolites including butyrate and medium-chain fatty acids were associated with some metabolites of the tryptophan-kynurenine pathway. Targeting the glutamatergic neurotransmission through the modulation of the kynurenine pathway, by manipulating the gut microbiota, might represent an interesting alternative for modulating alcohol-related behavior.

## Graphical abstract

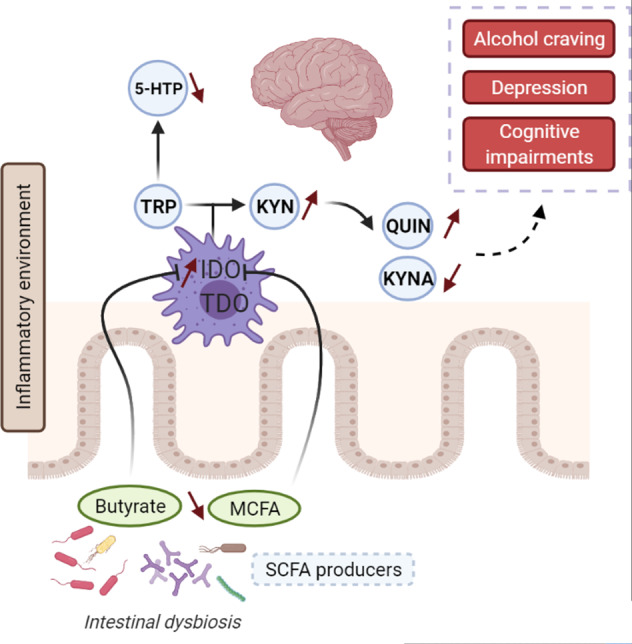

## Introduction

Alcohol use disorder (AUD) is a psychiatric disease associated with leaky gut and alterations of the gut microbiota [1, 2]. Numerous experimental and clinical studies have highlighted a link between intestinal bacteria and the presence of emotional and cognitive symptoms in multiple neurological and psychiatric conditions [3]. Animal studies have shed light on the complex mechanisms underlying gut-brain interactions which mainly include metabolic, immune and vagus nerve-dependent pathways. However, those communication pathways remain poorly studied in clinical populations. Our previous studies suggest that the degree of intestinal dysbiosis is related to the severity of alcohol dependence and that systemic inflammation could drive, at least in part, the emotional symptoms and alcohol craving [2, 4, 5]. However, the metabolic component of the gut-brain communication pathway has never been explored in AUD patients. Among the potentially circulating neuroactive metabolites, those arising from tryptophan metabolism have gained considerable attention since they are at the interface between intestinal bacteria, host immune response and brain functions [6].

**Fig. 1:**
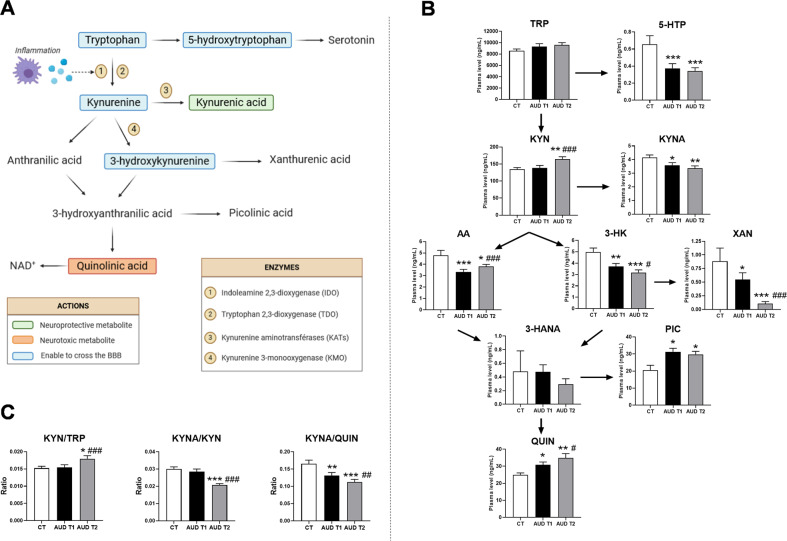
The tryptophan-kynurenine pathway and its related metabolic ratios. **A** The essential amino acid tryptophan (TRP) is the precursor of 5-hydroxytryptophan (5-HTP) which forms serotonin. However, the majority of peripheral TRP enters the kynurenine pathways thanks to two rate-limiting enzymes: the liver tryptophan 2,3-dioxygenase (TDO) and the extra-hepatic indoleamine 2,3-dioxygenase (IDO). The activity of IDO is negligible under basal conditions but dramatically inducible by inflammatory signals. Kynurenine (KYN) can be then metabolized through three distinct pathways to form kynurenic acid (KYNA), 3-hydroxykynurenine (3-HK) and anthranilic acid (AA) by the action of kynurenine aminotransferase (KAT), kynurenine 3-monooxygenase (KMO) and kynurenase enzymes, respectively. 3-HK can be further converted into xanthurenic acid (XAN), whereas both 3-HK and AA may be enzymatically converted to 3-hydroxyanthranilic acid (3-HANA), which in turn form picolinic acid (PIC) and quinolinic acid (QUIN). The final product of the pathway is nicotamine adenine dinucleotide (NAD + ), an important cofactor in cellular reactions linked to energy metabolism. **B** Plasma concentration of TRP and kynurenine metabolites of healthy controls (CT) and AUD patients at the onset (T1) and end (T2) of a 3-week detoxification program. **C** Ratio of metabolites reflecting the sum of IDO and TDO activities (KYN/TRP), KATs activity (KYNA/KYN) and the balance between neuroprotective and neurotoxic metabolites (KYNA/QUIN) in healthy controls and AUD patients during alcohol withdrawal. Results are expressed as mean ± SEM, *n* = 57 AUD patients and 16 CT subjects. *P < 0.05 vs. CT; **P < 0.01 vs. CT; ***P < 0.001 vs. CT; ^#^*P* < 0.05 vs. AUD T1; ^##^*P* < 0.01 vs. AUD T1; ^###^*P* < 0.001 vs. AUD T1.

The essential amino acid tryptophan (TRP) is the precursor of serotonin (5-hydroxytryptamine; 5-HT) which plays an important role in the central nervous system (CNS) and, in particular, on mood regulation. In mammals, more than 90% of TRP is degraded through the kynurenine pathway which generates a range of metabolites that regulate the immune response and neurotransmission thereby also impacting the CNS [7] (Fig. 1A). The conversion of TRP into kynurenine (KYN) is controlled by two rate-limiting enzymes, the liver tryptophan 2,3-dioxygenase (TDO) and the extra-hepatic indoleamine 2,3-dioxygenase (IDO), the activity of which is largely regulated by inflammatory stimuli such as cytokines, Toll-like receptors ligands, bacterial metabolites as well as bacteria-derived reactive oxygen species [8–13]. Of all the different by-products of the kynurenine pathway, the kynurenic acid (KYNA) and the quinolinic acid (QUIN) have been the most studied. KYNA is generally considered a neuroprotective and anticonvulsant metabolite. It is a recognized competitive antagonist of glutamate receptors (NMDA) and potentially of α7 nicotinic acetylcholine receptors (α7nAChR) [14, 15]. Fluctuation in brain KYNA has a direct impact on glutamatergic, dopaminergic and cholinergic neurotransmitter systems with behavioral and cognitive consequences, as shown in several neurological and psychiatric disorders [16]. By contrast, QUIN is an agonist of NMDA receptors causing glutamate release and seizure in mice after intracerebroventricular injection [17]. Other neurotoxic properties of QUIN are attributed to the generation of reactive oxygen species, depletion of endogenous antioxidants and lipid peroxidation [16, 18]. It is noteworthy that while some metabolites of the kynurenine pathway can easily enter the brain (Fig. 1A), KYNA and QUIN have very limited ability to cross the blood-brain barrier (BBB). An important question remains, especially for clinical studies, to understand whether the peripheral concentrations of those neuroactive metabolites could reflect their central levels susceptible to influence brain functions. Studies using pharmacological tools such as enzymatic inhibitors of the kynurenine pathway that do not cross the BBB showed similar changes in both plasma and brain levels of metabolites [19]. Another study showed a strong association between the levels of kynurenine metabolites measured in plasma and in the cerebrospinal fluid (CSF) in depressed patients [20]. Consequently, the association between peripheral metabolite concentrations and psychological or cognitive symptoms in clinical populations deserves to be investigated.

Studies exploring peripheral and central levels of TRP and kynurenine-related metabolites in AUD patients have led to discrepant results, linked to clinical population heterogeneity including the drinking status (actively drinking, short-term or long-term abstinence) of the patients [21]. Another crucial factor that has been neglected when studying the kynurenine pathway in AUD patients is the influence of the gut microbiota composition. Because tryptophan metabolism is at the interface between the intestinal bacteria and the host [6], it is tempting to speculate that alterations of the microbial composition might contribute to fluctuating levels of TRP and kynurenine metabolites with consequences for brain functions and behavior in AUD patients.

In this study, we proceeded in a stepwise manner to address several objectives. First, we assessed the circulating levels of TRP and kynurenine metabolites in AUD patients at the onset (T1) and end (T2) of a 3-week detoxification program, and compared them to those obtained in healthy controls. Second, we tested correlations between circulating metabolites, inflammatory markers and psychological symptoms of AUD patients. Third, we evaluated the overall influence of intestinal bacteria and bacterial metabolites on circulating TRP and kynurenine metabolites concentrations.

## Methods and material

### Subjects and study design

The 57 patients included in this study belong to an existing cohort of inpatients suffering from alcohol use disorder (AUD) and hospitalized for alcohol detoxification in the alcohol withdrawal unit of Saint-Luc academic Hospital, Brussels, Belgium [2, 4]. Since the study patients were enrolled before the publication of the DSM 5, they were therefore diagnosed as alcohol-dependent by a psychiatrist (PdT) using the criteria of the DSM-IV. Exclusion criteria were the following: the use of antibiotics, probiotics, glucocorticoids, or nonsteroidal anti-inflammatory drugs within 2 months preceding enrollment, the presence of metabolic disorders such as diabetes and obesity (body mass index >30 kg/m^2^), chronic inflammatory diseases (e.g., inflammatory bowel disease or rheumatoid arthritis), cancer, bariatric surgery, or other severe medical conditions, including significant liver fibrosis (fibrosis ≥F2 on transient liver elastography). Patients were admitted for a 3-week detoxification program. Patients were actively drinking until at least 24 h before admission. Patients who relapsed during the detoxification program were excluded from the study. Fasting blood was drawn from the antecubital vein on the day after admission (T1) and at the end of the detoxification program, after 18 days of alcohol abstinence (T2). AUD patients were compared to a control group of 16 healthy subjects (CT) who were matched for age, sex and BMI and who consumed socially low amounts of alcohol (<20 g/day). The study protocol was approved by the ethical committee of the hospital (B40320096274) and written informed consent were obtained from all subjects.

### Measurement of tryptophan metabolites

The TRP metabolites were measured by a previously described HPLC method [22]. Briefly, analytes were extracted from samples and calibrators/controls using Waters Oasis MCX extraction cartridges (Waters, Milford, MS). The eluent was then evaporated to dryness and reconstituted with 0.1 M PBS for injection into the HPLC system. Analyses were carried out on a Waters 2695 chromatograph with a 250 mm × 4 mm Supersphere 60 RP-select B, C8 column (Merck, Darmstadt, Germany) connected to a Waters 2487 dual-λ UV detector and a 2475 fluorescence detector. TRP (λex: 300 nm; λem: 350 nm) and 5-HIAA (λex: 300 nm; λem: 340 nm) were measured by fluorescence detection; KYN (365 nm), KYNA (330 nm), and 3-HK (365 nm) were measured by UV detection. Data were processed using EMPOWER software (Waters). The concentrations were established through comparison of peak heights of the single analytes with the peak heights of the respective calibration curves, always including the internal standard into the calculation. The method has been validated showing good absolute recovery and precisions (e.g. for 3-HK: recovery of 85,8%, intra-day precision of 3.9%, and inter-day precision of 7.5%).

### Measurement of inflammatory markers: hsCRP and cytokines

Plasma hsCRP was measured by an automated turbidimetry method (DxC 800, Beckman Coulter). TNFα, IL-6 and IL-10 have been measured by the multiplex immune assay as described previously [4].

### Measurement of cortisol

Cortisol was measured in the saliva, as the salivary levels closely reflect the serum levels of unbound (free) and biologically active forms of cortisol [23]. Saliva samples were collected in the afternoon (between 2 and 4 pm) using the Sarstedt Salivette collection devices (Nümbrecht, Germany), and stored at −20 °C. The cortisol assays were carried out at the Department of Clinical Biochemistry, in St-Luc academic hospital (Brussels, Belgium). Saliva was extracted from the cotton swab by centrifugation (1000 g, 2 min) and the cortisol was measured using a competitive polyclonal immunoassay, comprised of an electromagnetic separation step followed by electrochemiluminescence quantitation with the Elecsys 1010/2010 analyser (Roche Diagnostics, Mannheim, Germany). The intra- and interassay coefficients were, respectively, 4.0 and 7.2%.

### Assessment of psychological symptoms

Emotional symptoms related to depression, anxiety and alcohol craving were evaluated using self-reported questionnaires, namely the Beck Depression Inventory (BDI), the State-Trait Anxiety Inventory (STAI) and the Obsessive-Compulsive Drinking Scale (OCDS) as reported previously [4]. Selective attention was evaluated with the validated simple binary computerized task from the “Batterie d’Attention de William Lennox” (BAWL) in its version 4.0. Briefly, the subject was asked to react when a specific target appeared on the screen by pressing as quickly as possible the response button on the computer. Reaction time (in ms) needed to press the button is recorded.

### Gut microbiota analysis

Fecal samples were collected, in a subset of 13 AUD patients at T2 and 14 healthy controls, in a sterile container and immediately stored at −80 °C until further processing. Bacterial DNA extraction was performed by using the repeated bead-beating procedure with a modified protocol for the QIAamp Stool DNA Mini Kit (Qiagen). 454 pyrosequencing of the 16 S rDNA gene was conducted and data (comparison between AUD and CT) have been reported in our previous paper [2]. The taxonomic level ‘genus” was considered in this study for the rCCA. Quantification of *Faecalibacterium prausnitzii* was performed by qPCR as reported [2]. The relative quantification of short-chain fatty acids and medium-chain fatty acids was performed in the fecal samples on a gas chromatography-mass spectrometry (GC-MS) quadrupole as already described [2].

### Statistical analysis

Results were analyzed using SPSS V.25 and the package MixOmics in R 4.0 [24]. Graphs and figures were drawn with GraphPad Prism 8, Cytoscape 3.8.0 and the web-based software BioRender. Regarding the analysis of the tryptophan pathway, after logarithmic transformation, unpaired t-tests and paired t-tests were used to compare AUD to CT, and AUD T1 to AUD T2, respectively. When logarithmic transformation did not allow to normalize the distribution of the variables, the results were analyzed using non-parametric tests (Mann-Whitney for unpaired tests and Wilcoxon for paired tests). Data presented in graphs are non-transformed means ± SEM or SD as indicated in the figure legends. Statistical significance was defined as *P* <0.05. The multivariate statistical approach “regularized canonical correlation analysis” (rCCA) was conducted with the R package MixOmics to integrate two omics data sets related to the 16 S rDNA metagenomics and metabolomics [25]. Graphical outputs such as correlation circle plot, relevance networks and clustered image map were used to visualize the correlation structure between both biological data sets. The investigators were not blinded during data acquirement and analysis. Sample size cannot be calculated here as the effect size and the inter-individual variability are unknown. This study is considered as exploratory and the current number of patients is based on similar studies [2, 4, 5].

## Results

### AUD is associated with lower level of neuroprotective KYNA and higher level of neurotoxic QUIN

The biological and psychological features of AUD patients and healthy controls (CT) are described in Table 1. Both populations were matched for sex, age and body mass index. AUD patients presented with higher hsCRP levels, higher scores of depression, anxiety and alcohol craving and lower reaction time at a selective attention task compared to healthy subjects. The plasma concentrations of TRP and kynurenine metabolites of CT subjects and of AUD patients tested at onset (T1) and end (T2) of a 3-week detoxification program are depicted in Fig. 1B. The levels of plasma TRP did not differ between AUD patients and CT subjects. TRP can either be metabolized through the serotonin or the kynurenine pathway. We found that 5-hydroxytryptophan (5-HTP), the direct precursor of serotonin, was significantly decreased in AUD patients at both times of alcohol withdrawal. The central metabolite of the kynurenine pathway, KYN, was increased in AUD patients only at the end of the detoxification program (T2). The neuroprotective metabolite KYNA was lower in AUD patients than in CT, the effect persisting after alcohol withdrawal. KYN can also be converted into anthranilic acid (AA) and 3-hydroxy-kynurenine (3-KH) which were both decreased in AUD patients. The decrease in 3-HK was accompanied by a reduction of plasma xanthurenic acid (XAN), even more importantly at T2. The detection of the metabolite 3-hydroxy-anthranilic acid (3-HANA) varies among individuals across the groups, since it was quantified in 19% of CT, 50% of AUD at T1 and 35% of AUD at T2. The levels of picolinic acid (PIC) were higher in AUD patients compared to CT. Finally, the neurotoxic metabolite QUIN was significantly higher in AUD patients at both times of withdrawal compared to CT.

**Table 1:** Biological and psychological features of AUD patients and healthy controls.

	CT	AUD T1	AUD T2	*P-value (CT* vs. *AUD T1)*
**N**	16	57	57	
**Sex (n) (% Male)**	9 M/7 F (56%)	41 M/16 F (72%)	41 M/16 F (72%)	**0.23**
**Age (years)**	50 ± 11	49 ± 10	49 ± 10	**0.81**
**BMI (kg/m**^**2**^**)**	26.3 ± 3.1	24.8 ± 4.6	24.9 ± 4.5	**0.25**
**Alcohol consumption (g/day)**	10 ± 7	181 ± 91	0	**<0.001**
**hsCRP (mg/dL)**	0.12 ± 0.07	0.38 ± 0.64	0.41 ± 0.61	**0.007**
**Depression score (BDI)**	5 ± 5	28 ± 11	14 ± 11	**<0.001**
**State Anxiety score (STAI)**	28 ± 8	46 ± 13	38 ± 12	**<0.001**
**Alcohol craving score (OCDS)**	1 ± 1	21 ± 5	6 ± 6	**<0.001**
**Selective attention – reaction time (ms)**	388 ± 65	487 ± 100	432 ± 67	**<0.001**

To better assess the effect of alcohol withdrawal on the kynurenine pathway, we calculated various ratios of metabolite concentration that have been largely utilized as indicators of enzymatic activities [26]. We found that the KYN-to-TRP ratio reflecting the sum of IDO and TDO activities was increased whereas the KYNA-to-KYN ratio reflecting KATs activity was decreased upon withdrawal (Fig. 1C). This suggests that 3 weeks of alcohol abstinence activates IDO/TDO and worsened the imbalance between neuroprotective and neurotoxic metabolites (KYNA-to-QUIN ratio) (Fig. 1C).

Overall, the metabolite concentrations did not differ between male and female AUD patients, at T1 or T2, except for the level of plasma TRP which was higher in men compared to women at T2 only (Fig S1A). In CT subjects, 3-HK and QUIN were both higher in women (Fig S1 B, C). Interestingly, no correlation was found between the metabolites and the amount of alcohol consumed.

Altogether, these results showed a shift from the serotonin pathway to the kynurenine pathway in AUD patients, leading to an increase in the neurotoxic metabolite QUIN and to a reduced level of neuroprotective KYNA. A 3-week detoxification program induced a significant elevation of KYN, reinforce the drop in 3-HK and XAN and the increase in QUIN.

### Tryptophan and kynurenine metabolites are associated with inflammation and with psychological symptoms

**Fig. 2:**
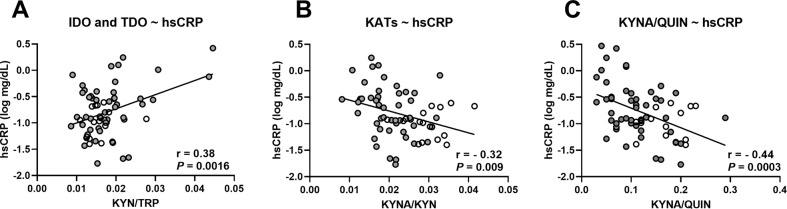
Correlations between the inflammatory markers and the metabolic ratios. Correlations between the plasma level of inflammatory markers hsCRP and the metabolic ratio reflecting (**A**) the sum of IDO and TDO activities, (**B**) KATs activity and (**C**) the balance between neuroprotective and neurotoxic metabolites. The correlations were calculated in healthy controls (white circles) and in AUD patients (gray circles) at the end of the detoxification program (T2) using Pearson’r coefficient, *n* = 65.

The switch from the serotonin to the kynurenine pathway is enhanced in inflammatory conditions since IDO is particularly inducible by inflammatory stimuli. Indeed, we found a positive correlation between the inflammatory marker hsCRP and the KYN-to-TRP ratio (Fig. 2A). In addition, hsCRP was negatively correlated with KATs activity which regulates the production of neuroprotective metabolite (Fig. 2B), as well as with the KYNA-to-QUIN ratio (Fig. 2C). These correlations, obtained at the end of the 3-week detoxification program (T2), suggest that inflammation activates the kynurenine pathways and induces a metabolic switch towards the neurotoxic arm. It is worth noting that specific inflammatory cytokines, TNFα, IL-6 and IL-10 did not correlate with the KYN-to-TRP ratio. However, those cytokines were positively correlated with the neurotoxic metabolites QUIN and negatively correlated with the KYNA-to-QUIN ratio (Table S1). The KYN-to-TRP ratio also reflects liver TDO activity which is enhanced by glucocorticoids. We therefore measured cortisol levels in the saliva and found that they were higher in AUD at T1 compared to healthy subjects, and decreased during alcohol withdrawal (*P* = 0.07) (Fig S2). At T1, cortisol levels were significantly correlated with the metabolic ratios KYN-to-TRP, KYNA-to-KYN and KYNA-to-QUIN (Fig S3 A–C).

**Fig. 3:**
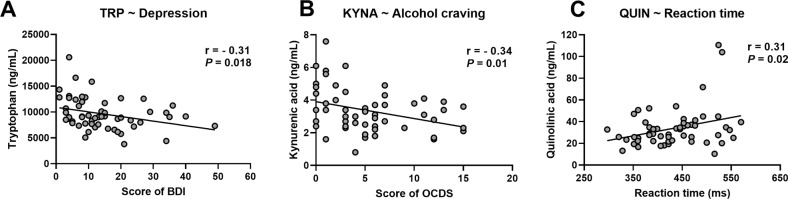
Correlations between the tryptophan metabolites and the psychological symptoms. Correlations between the plasma level of TRP and kynurenine metabolites (KYNA and QUIN) and the score of (**A**) depression, (**B**) alcohol craving, and (**C**) the reaction time measured during a selective attention task. The correlations were calculated in AUD patients at the end of the detoxification program (T2) using Pearson’r coefficient, *n* = 56.

Since the kynurenine metabolites are known to be neuroactive by influencing brain neurotransmission, we tested whether they were correlated with psychological or cognitive symptoms developed by AUD patients. We found that, at the end of the detoxification program (T2), TRP was negatively correlated with the score of depression and KYNA was negatively correlated with alcohol craving (Fig. 3A, B). The neurotoxic metabolite QUIN was associated with a slower reaction time at a selective attentional task (Fig. 3C). No correlation was found with the score of anxiety.

### Specific bacteria and bacterial metabolites are linked to the circulating levels of kynurenine metabolites

**Fig. 4:**
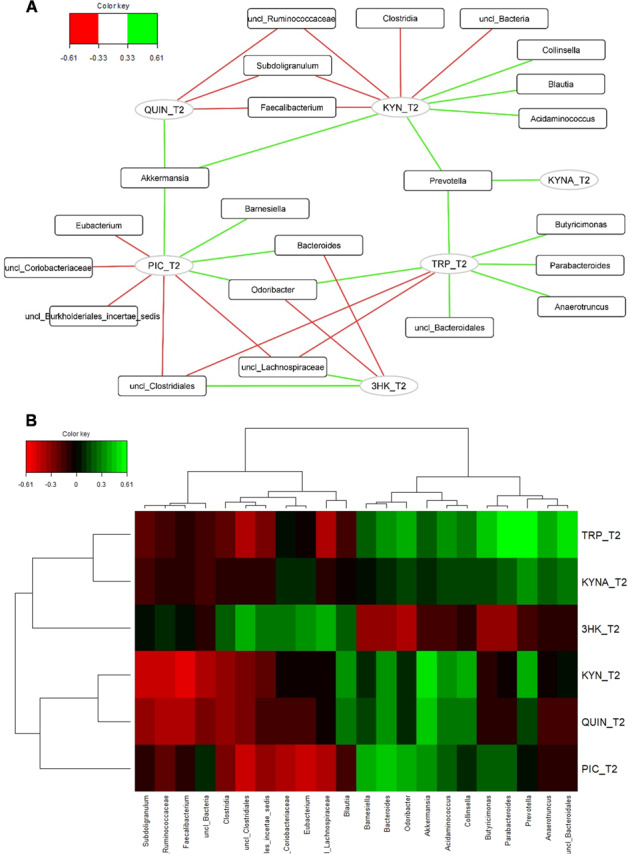
Associations between tryptophan metabolites and intestinal bacteria. **A** Relevance networks obtained for the first three dimensions of the rCCA and generated with Cytoscape. Green and red edges indicate positive and negative correlations respectively, with a threshold = 0.33. Tryptophan metabolites and intestinal bacteria are represented respectively as circles and rectangles. **B** Clustered Image Map obtained for the first three dimensions of the rCCA. The green and the red colors indicate positive and negative correlations respectively, with a threshold = 0.33, whereas black indicated small correlation values.

Since tryptophan metabolism is at the interface between the intestinal bacteria and the host, we tested whether intestinal bacteria could be linked to the levels of circulating kynurenine metabolites. For this purpose, we conducted regularized canonical correlation analysis (rCCA) on the 16 S rDNA gene sequencing data (considering the genus taxonomic level) and the plasma levels of tryptophan metabolites obtained at T2. This analysis performed pair-wise associations between the two omics data sets. The 3 graphical outputs of the rCCA, namely the correlation circle plot, the relevance network, and the clustered image map, are depicted in Figs. 4 and S4. The relevance network revealed that the metabolites TRP, KYN, 3-HK, KYNA, QUIN, and PIC were associated with a total of 22 bacterial taxa (Fig. 4A). Some bacteria like *Prevotella*, *Akkermansia*, *Faecalibacterium*, *Subdoligranulum*, *Bacteroides,* and *Odoribacter* were correlated with several metabolites of the tryptophan pathway. Among the strongest associations (r >0.4) (Fig. 4B), we found that TRP was positively associated with *Anaerotruncus*, *Butyricimonas*, *Parabacteroides*, *Prevotella,* and *Odoribacter*. KYN was negatively associated with members of the Ruminococcaceae family such as *Faecalibacterium* and *Subdoligranulum* and positively associated with *Akkermansia*. Interestingly, the neurotoxic metabolite QUIN was negatively correlated with *Faecalibacterium* but positively associated with *Akkermansia*. The level of PIC was positively associated with *Bacteroides* and *Barnesiella* and negatively correlated with *Eubacterium*. The neuroprotective metabolite KYNA was positively correlated with *Prevotella* but this association was weak (r<0.4).

**Fig. 5:**
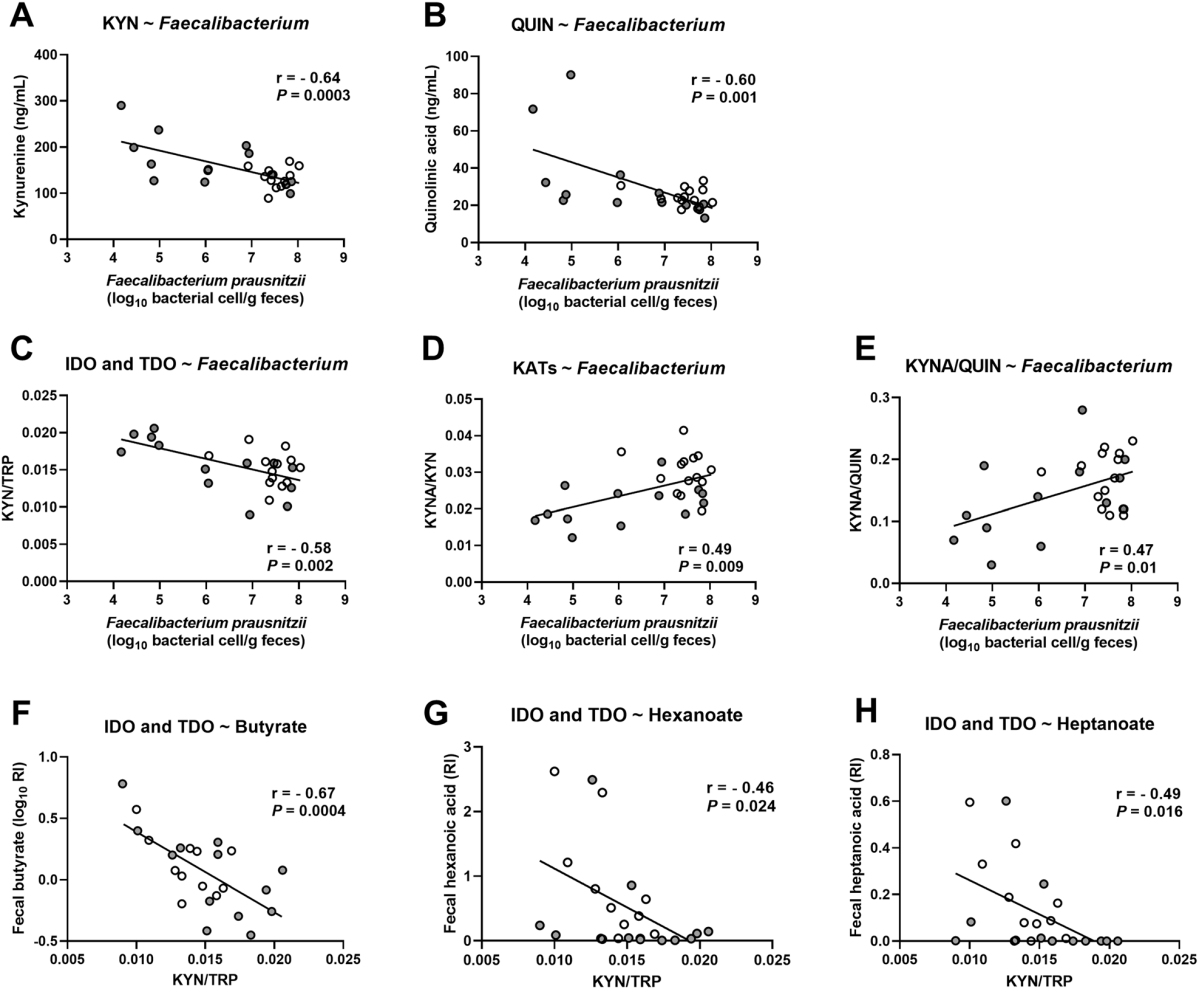
Correlations between tryptophan metabolites, intestinal bacteria and bacterial metabolites. Correlations between the levels of *F.prausnitzii* measured by qPCR and the metabolites (**A**) KYN, (**B**) QUIN and with the metabolic ratio reflecting (**C**) IDO and TDO activities, (**D**) KATs activity and (**E**) the balance between neuroprotective and neurotoxic metabolites. **F**–**H** Correlations between the fecal levels of short-chain- and medium-chain fatty acids and the KYN-to-TRP ratio reflecting IDO and TDO activities. The correlations were calculated in healthy controls (white circles) and in AUD patients (gray circles) at the end of the detoxification program (T2) using Pearson’r coefficient, *n* = 24–27.

Because inflammatory stimuli are important activators of the kynurenine pathway, we tested whether *Faecalibacterium prausnitzii*, known for its anti-inflammatory properties, could be related to kynurenine metabolites and to enzymatic activities of the pathway. More accurate quantification of the level of *F. prausnitzii* was obtained by qPCR as described in our previous paper [2]. We confirmed that *F. prausnitzii* was negatively correlated with KYN and QUIN, as reported in the relevance network, and also with IDO and TDO activities (Fig. 5A–C). We found positive associations between *F. prausnitzii* and KATs activity and with the ratio between neuroprotective and the neurotoxic metabolite (KYNA-to-QUIN ratio) (Fig. 5D, E).

Some of the bacteria highlighted in the relevance network are producers of short-chain fatty acids (SCFAs) such as butyrate which exerts an anti-inflammatory effect. Here, we found that fecal butyrate levels were similar between AUD and CT, while levels of medium-chain fatty acids (MCFAs) such as hexanoate and heptanoate were lower in AUD at T2 compared to CT (Fig S5A–C). Interestingly, butyrate, hexanoate and heptanoate were negatively correlated with IDO and TDO activity (Fig. 5F–H).

## Discussion

The metabolite TRP has been studied for a long time in the field of psychiatry since it is the precursor of serotonin, an important neuromodulator that contributes to depression symptomatology. However, more recent data suggest that downstream metabolites of TRP catabolism belonging to the kynurenine pathway, due to their neurotoxic potential, may also play a key role in the development of various neurological and psychiatric disorders [16]. TRP metabolism is greatly influenced by acute and chronic alcohol consumption and subsequent withdrawal in both humans and experimental animals [21, 27]. In the present study, we showed, in a well-characterized population of AUD patients tested at the onset and end of a 3-week detoxification program in highly controlled and standardized clinical settings, that gut microbiota composition, systemic inflammation and psychological symptoms were associated with the plasmatic concentrations of TRP metabolites.

First, we show that the levels of TRP were similar in healthy subjects and in AUD patients before and after the detoxification process whereas the concentration of KYN raised after 3 weeks of alcohol withdrawal. Our data are consistent with other studies showing constant TRP levels during withdrawal [28] and higher KYN concentration in 1-month abstinent alcoholics than during the first week of detoxification [29]. Some studies also showed that KYN concentration remained high in subjects who maintained abstinence while it decreased in subjects who relapsed into drinking [29]. They further suggested that liver damage induced by active alcohol consumption could inhibit hepatic TDO activity, which is therefore enhanced with prolonged abstinence. However, it is possible that a higher KYN concentration observed in abstinent AUD subjects is also due to the persistence of inflammation at the end of the detoxification [4]. This is supported by a higher KYN-to-TRP ratio, reflecting both IDO and TDO activities, and a positive correlation between IDO and TDO activities and the inflammatory marker hsCRP observed at the end of alcohol withdrawal. Some authors also suggest that the persistence of enhanced KYN-to-TRP ratio after several weeks of abstinence may be partly responsible for the persistence of depressive symptomatology in detoxified alcoholics [28], which is consistent with elevated blood KYN-to-TRP ratio in patients with depression [30]. Here, we found that circulating TRP was negatively correlated with depression score in AUD patients, as it has already been reported in patients with major depression [31]. Altogether, these results suggest that the persistence of inflammation in detoxified AUD patients leads to IDO/TDO activation and elevated KYN levels, which are accompanied by the persistence of depression. How systemic IDO activation leads to depressive behavior is currently unclear but immunohistochemical studies of post-mortem brain of patients with major depression have revealed microglial activation with QUIN immunoreactivity and reduction of the number of astrocytes, the glial cells responsible for KYNA production [32]. Consistent with that, we found elevated systemic QUIN and decreased KYNA in AUD patients at both times of withdrawal.

The glutamatergic system has received great attention with respect to its role in addictive behaviors, such as drug-seeking and relapse-like behavior, and both metabolites, KYNA and QUIN, can modulate glutamate neurotransmission. Two recent studies in rodents showed that KMO inhibition, which induces a peripheral and central metabolic switch of the kynurenine pathway towards the production of KYNA, reduced alcohol-seeking and relapse behavior in rats [33] and decreased ethanol consumption and preference in mice [19]. Interestingly, we found in the present study that KYNA was negatively associated with the intensity of alcohol craving in AUD patients at the end of the detoxification program, which represents a critical period where patients are prone to relapse. The effects of KYNA on alcohol-related behaviors might be explained by several mechanisms. For instance, animal studies have shown that KYNA induces aversion to ethanol by inhibiting the liver mitochondrial aldehyde dehydrogenase (ALDH) activity which results in increased concentration of acetaldehyde, a metabolite that causes immediate aversive symptoms towards alcohol [34].

Since the production of acetaldehyde requires the presence of ethanol, this mechanism seems unlikely in this present study for the correlation between KYNA and craving that was obtained at the end of 3-week alcohol abstinence. Then, several lines of evidence attribute anti-inflammatory properties of KYNA by the activation of AhR [14], while peripheral and central inflammation has been extensively linked to alcohol drinking behavior [5, 35]. Additionally, KYNA modulates the reward system by acting on the α7nAChR which regulates the extracellular levels of glutamate that in turn modulates dopamine release in the nucleus accumbens [19]. Finally, the metabolite QUIN was associated with a slower reaction time at the attentional task in AUD patients. Although this correlation may be coincidental, it can not be excluded that it could be due to the excitotoxic properties of this metabolite. A recent study showed a high correlation between plasma and CSF QUIN levels and found that peripheral inflammation could mediate this association. Here, we found that plasma QUIN was positively correlated with inflammatory cytokines TNFα and IL-6. This may suggest that peripheral inflammation leads to increased brain levels of QUIN that in turn locally produces inflammatory cytokines that have a deleterious impact on cognition. Studies investigating the link between QUIN and cognitive deficits in psychiatric diseases are scare, although its level is increased in various cognitive disorders such as dementia, which is also characterized by an inflammatory component [36], Alzheimer’s disease [37] and Huntington’s disease [38]. Interestingly, a study found that more severe cognitive impairments in schizophrenic patients were correlated to increased plasma levels of quinolinic acid [39].

Recently, it has been demonstrated that the TRP metabolic pathways are under the direct or indirect influence of intestinal bacteria and their metabolites [6, 40, 41]. For instance, germ-free and antibiotic-treated animals have altered levels of circulating TRP, KYN and 5-HT which can be normalized post-colonization [42–44]. The production of 5-HT by enterochromaffin cells of the gut is regulated by spore-forming bacteria like Clostridiales and SCFAs [45–47]. In the present study, we explored the potential associations between bacteria and bacterial metabolites with the plasma levels of TRP metabolites. First, we found that circulating TRP was positively correlated with several bacteria known as SCFA producers, such as *Prevotella*, *Odoribacter*, *Butyricimonas*, *Parabacteroidetes*, *Anaerotruncus* [48–52]. SCFAs, like acetate, propionate and butyrate, are important metabolites maintaining intestinal homeostasis, strengthening the gut barrier and exerting immunomodulatory functions [53]. Bacteria that ferment fibers and produce SCFAs are typically reduced in feces of patients with inflammatory bowel diseases (IBD). Secondly, we found that butyrate-producers of the Ruminococcaceae family such as *Faecalibacterium* and *Subdoligranulum* were negatively correlated with KYN. We therefore hypothesized that TRP and KYN levels might be influenced by bacteria or bacterial metabolites with anti-inflammatory properties. Indeed, we found that the KYN-to-TRP ratio (reflecting IDO and TDO activities) was negatively correlated with the anti-inflammatory bacterium *F. prausnitzii* but also with the fecal levels of butyrate, hexanoate and heptanoate. Those data are in line with previous studies showing the potent anti-inflammatory actions of butyrate and MCFA through the down-regulation of IDO and the activation of peroxisome proliferator-activated receptor (PPAR)-gamma, respectively [9, 54]. Also, MCFA is the most discriminatory metabolites in IBD, with lower fecal levels in patients with Crohn disease and ulcerative colitis compared to healthy subjects [55]. Interestingly, *F. prausnitzii* is reduced in IBD [56] but also in multiple psychiatric conditions, such as major depression [57, 58], autism [59], AUD [2, 60, 61] and bipolar disorder [62]. Intriguingly, we found in the present study, that the neurotoxic metabolite QUIN was negatively and positively associated with *F. prausnitzii* and *Akkermansia*, respectively. Although *Akkermansia* has beneficial effects in the context of obesity and metabolic disorders [63], its role in neurological and psychiatric diseases is more controversial with elevated abundance in the feces of patients with autism [59, 64], multiple sclerosis [65, 66], Parkinson’s disease [67, 68] and schizophrenia for which an association with QUIN has already been observed [69].

Our study presents several limitations. First, it relies only on peripheral metabolite concentrations. Although some metabolites can easily enter the brain, such as TRP, KYN, 3-HK, the neuroactive metabolites KYNA and QUIN have very limited ability to penetrate the blood-brain barrier (BBB) and must therefore be produced locally within the brain. Furthermore, the central availability of TRP mainly depends on the competition by the large amino acids (tyrosine, valine, leucine, isoleucine, phenylalanine) at the transport across the BBB but the circulating level of those competing amino acids were not measured in the present study. It is therefore a major challenge especially in clinical studies to understand how peripheral metabolites concentrations can influence brain functions. Some studies using enzymatic inhibitors that do not cross the BBB showed a similar impact on both peripheral and central levels of kynurenine metabolites [19]. Another recent study showed a strong association between the levels of kynurenine metabolites measured in plasma and in the cerebrospinal fluid (CSF) in depressed patients [20], arguing in favor of the clinical relevance of testing the association between peripheral metabolite concentrations and psychological symptoms. In addition, neurological and psychiatric conditions, such as AUD, can be associated with a leaky BBB that could facilitate the translocation of the neuroactive metabolites [70]. The second limitation is linked to the role played by SCFA producers in the fluctuation of TRP metabolites. The abundance of these bacteria, such as *Prevotella* is influenced by dietary habits [71], especially the consumption of carbohydrates, which have not been assessed in this study.

In conclusion, we showed an activation of the kynurenine pathways in AUD patients and a switch towards the neurotoxic arm with increased peripheral levels of QUIN and decreased concentrations of KYNA, particularly at the end of a 3-week detoxification program. While numerous preclinical studies have shown an overall influence of the gut microbiota on TRP and KYN metabolism [72], also in the context of alcohol consumption [73], evidence in humans is lacking. We show, in the present study, that SCFA producers, such as *Faecalibacterium prausnitzii*, and the metabolites butyrate and MCFA that exert anti-inflammatory effects might play a role in the regulation of the TRP metabolic pathway. We also show for the first time that the neuroprotective metabolite KYNA, which modulates glutamatergic neurotransmission, was negatively correlated with the score of alcohol craving. While current pharmacological drugs targeting the NMDA receptors showed only modest efficacy and are accompanied by multiple side effects [74], an indirect approach targeting glutamatergic neurotransmission through the modulation of the kynurenine pathways might represent an interesting alternative for modulating alcohol-related behavior.

## Supplementary information

Supplemental figures and tables
