# Supplementary material for: Alterations of kynurenine pathway in alcohol use disorder and abstinence: a link with gut microbiota, peripheral inflammation and psychological symptoms
Source: Transl Psychiatry. 2021 Oct 1;11:503. doi: 10.1038/s41398-021-01610-5 (PMC8486842; doi:10.1038/s41398-021-01610-5)
Supplement: Supplementary file 1 — Supplemental figures and tables [file 41398_2021_1610_MOESM1_ESM.docx]

**Supplemental figures**

**Figure S1: Effect of sex on tryptophan metabolites**

Fig S1: Differences in males and females were observed for the plasma levels of metabolites TRP, 3-HK and QUIN either in AUD patients or in CT subjects. Results are expressed as mean ± SEM. * *P* < 0.05

**Figure S2: Salivary cortisol levels**


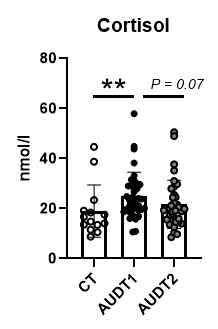


Fig S2: Concentrations of cortisol measured in saliva samples of healthy controls (CT) and AUD patients at the onset (T1) and end (T2) of alcohol withdrawal. Results are expressed as mean ± SD. ** *P* < 0.01

**Figure S3: Association between salivary cortisol and the metabolic ratios**

Fig S3: Correlations between salivary cortisol levels and the metabolic ratios reflecting (A) the sum of IDO and TDO activities, (B) KATs activity and (C) the balance between neuroprotective and neurotoxic metabolites. The correlations were calculated in healthy controls (white circles) and in AUD patients (black circles) at the onset of the detoxification program (T1) using Pearson’r coefficient, n = 47.

**Figure S4: Correlation circle plot**


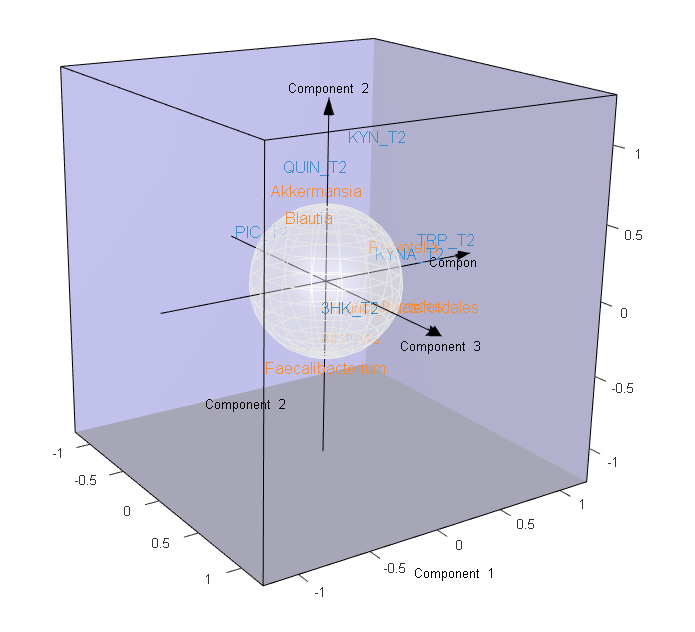


Fig S4: Correlation circle plot for the first three dimensions (components) of the rCCA, with a threshold = 0.5.

**Fig S5: Fecal levels of short chain and medium chain fatty acids**


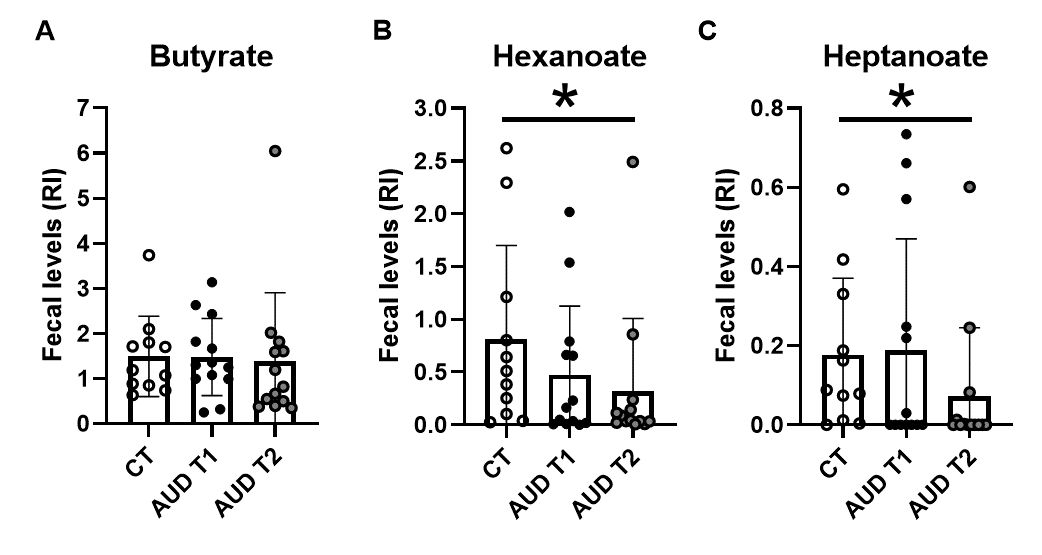


Fig S5: Measurement of the short chain fatty acid butyrate and medium chain fatty acids hexanoate and heptanoate in the feces of healthy controls (CT) and AUD patients at the onset (T1) and end (T2) of alcohol withdrawal. Results are expressed as mean ± SD. * *P* < 0.05

**Table S1: Correlation between inflammatory cytokines, quinolinic acid and metabolic ratios at the end of alcohol withdrawal.**

|  | QUIN | KYN/TRP | KYNA/QUIN |
| --- | --- | --- | --- |
| TNFα | R= 0.332  *P* = 0.028 | R = 0.229  *P* = 0.135 | R = -0.370  *P* = 0.014 |
| IL-6 | R= 0.595  *P* = 0.001 | R = 0.075  *P* = 0.705 | R = -0.544  *P* = 0.003 |
| IL-10 | R= 0.337  *P* = 0.029 | R = -0.017  *P* = 0.917 | R = -0.050  *P* = 0.753 |
